# Lupeol as a Potential Inhibitor of NorA Efflux Pumps in *Staphylococcus aureus*: In Silico and In Vitro Evidence

**DOI:** 10.1002/open.202500227

**Published:** 2025-07-22

**Authors:** Nara Juliana Santos Araújo, Camila Aparecida Pereira Silva, Vanessa Lima Bezerra, Cicera Datiane Morais Oliveira‐Tintino, Gabriel Gonçalves de Alencar, Maria do Socorro Costa, Ana Raquel Pereira da Silva, Josefa Sayonara dos Santos, Kamila Correa Camara, Heberty diTarso Fernandes Facundo, Lívia Pereira Ferreira, Henrique Douglas Melo Coutinho, José Maria Barbosa Filho, Carolina Bandeira Domiciano, José Bezerra de Araújo‐Neto, Jacqueline Cosmo Andrade‐Pinheiro

**Affiliations:** ^1^ Graduate Program in Biochemistry and Molecular Biology Federal University of Cariri Barbalha Ceará 63.090‐684 Brazil; ^2^ Laboratory of Applied Microbiology‐LAMAP Federal University of Cariri Barbalha Ceará 63.090‐684 Brazil; ^3^ Graduate Program in Health Sciences Federal University of Cariri Barbalha Ceará 63.090‐684 Brazil; ^4^ Department of Biological Chemistry Regional University of Cariri Crato Ceará 63.105‐010 Brazil; ^5^ Laboratory of Microbiology and Molecular Biology Regional University of Cariri ‐ LMBM Crato Ceará 63.105‐010 Brazil; ^6^ Faculty of Medicine Federal University of Cariri Barbalha Ceará 63.090‐684 Brazil; ^7^ Institute of Research in Drugs and Medicines Federal University of Paraiba ‐ UFPB João Pessoa Paraíba 50.051‐900 Brazil; ^8^ Graduate Program in Biological Sciences Biosciences Center Federal University of Pernambuco Recife Pernambuco 50.670‐901 Brazil

**Keywords:** docking moleculars, emissão fluorescência, *NorA*, permeabilidade de membranes, *Staphylococcus aureus*

## Abstract

Antimicrobial resistance remains one of the major challenges to global public health, compromising the effectiveness of treatments and contributing to increased morbidity and mortality associated with bacterial infections. Among the mechanisms involved, efflux pumps—such as NorA, expressed in resistant strains of *Staphylococcus aureus*—are particularly noteworthy. These transport proteins actively expel antibiotics from the cell, reducing their intracellular concentration. In this context, natural compounds have been explored as potential resistance inhibitors, with a focus on the triterpene lupeol, known for its pharmacological properties. This study evaluates the activity of lupeol against the NorA efflux pump using in vitro assays and in silico modeling. The minimum inhibitory concentration (MIC) is determined by broth microdilution, and pump inhibition is assessed via ethidium bromide‐induced fluorescence. SYTOX Green assays indicated that lupeol does not compromise bacterial membrane integrity. Although lupeol presented a MIC ≥ 1024 μg mL^−1^, it demonstrates significant inhibition of NorA activity. Molecular docking reveals a binding energy of –7.112 kcal mol^−1^ and interactions with key residues of the protein, outperforming the CCCP control. These findings suggest that lupeol acts as a modulator of bacterial resistance, with potential application as a therapeutic adjuvant in the treatment of infections caused by multidrug‐resistant *S. aureus.*

## Graphical abstract

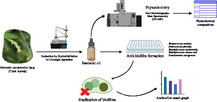

## 1 Introduction

*Staphylococcus aureus* is a multidrug‐resistant pathogen widely responsible for human infections, ranging from skin and soft tissue infections to more severe conditions such as pneumonia, endocarditis, and sepsis, often occurring in hospital settings. Antimicrobial resistance is a growing concern, as *S. aureus* has developed efficient mechanisms to evade therapeutic effects, one of the most prominent being the expression of efflux pumps such as NorA. These pumps act by expelling antibiotics from the cell, reducing their intracellular concentrations and, consequently, treatment efficacy.^[^ ^1^ ^]^ NorA is an efflux pump belonging to the major facilitator superfamily and plays a central role in the resistance of *S. aureus* to fluoroquinolones and other antibiotics.^[^ ^2^, ^3^ ^]^ The identification and characterization of inhibitors targeting this pump are essential for the development of therapies aimed at reversing bacterial resistance.

Natural products derived from plants have emerged as promising alternatives for combating bacterial resistance, particularly by modulating the activity of efflux pumps. These bioactive compounds possess diverse chemical structures, which may contribute to the variety of their mechanisms of action. Many of them have demonstrated the ability to inhibit efflux pump activity.^[^ ^4^ ^]^ Flavonoids, terpenes, and phytosterols, for example, have been identified as effective agents in modulating efflux pump‐mediated bacterial resistance.^[^ ^5^, ^6^ ^]^ However, many of these compounds still lack clinical studies to validate their therapeutic potential.

Among natural compounds, triterpenes have gained attention due to their wide range of biological activities, including antimicrobial, anti‐inflammatory, and antioxidant effects. Lupeol, a triterpene isolated from various plant species, has shown potential as an efflux pump inhibitor. It is known for its antimicrobial activity against several pathogens, including *S. aureus*, as well as for its anti‐inflammatory and antitumor properties.^[^ ^7^, ^8^ ^]^ In addition, other triterpenes, such as ursolic and oleanolic acids, have been studied for their inhibitory activity against efflux pumps. For example, ursolic acid has been shown to reduce NorA expression in *S. aureus*, suggesting a mechanism of action similar to that of lupeol, although with slightly lower efficacy.^[^ ^9^ ^]^ These triterpenes act not only by modulating efflux pump activity but also through effects on the bacterial membrane, contributing to increased intracellular concentrations of antimicrobials.^[^ ^10^ ^]^

The activity of lupeol is of particular interest because it appears to act more specifically and with lower toxicity compared to other compounds, such as CCCP (carbonylcyanide m‐chlorophenyl hydrazone), a known efflux pump inhibitor. Lupeol demonstrates greater affinity for NorA and fewer side effects.^[^ ^2^ ^]^ Experimental studies have shown that lupeol does not induce membrane permeability in bacterial cells, suggesting that its mechanism of action involves direct interaction with the efflux pump without compromising cellular integrity.^[^ ^8^ ^]^

This study aims to investigate the ability of lupeol to inhibit the NorA efflux pump in *S. aureus* and to compare its activity with that of other triterpenes, using both experimental and in silico approaches to better understand the underlying mechanisms of this inhibition. The goal is to provide a solid scientific foundation for the development of new treatments capable of more effectively combating resistant infections.

## 2 Results and Discussion

### 2.1 Determination of Minimum Inhibitory Concentration and Evaluation of Efflux Pump Inhibition

The evaluation of the minimum inhibitory concentration (MIC), performed using the broth microdilution method in 96‐well plates, showed that lupeol exhibited an MIC ≥ 1024 μg mL^−1^ against the tested *Staphylococcus aureus* strains. In contrast, Muktar et al.^[^ ^6^ ^]^ reported an MIC of 12.5 μg mL^−1^ using the agar well diffusion method. This discrepancy may result from methodological differences, as agar diffusion assays are less precise and not suitable for quantitative MIC determination, especially for hydrophobic compounds. Broth microdilution, recommended by CLSI and EUCAST, provides more accurate and reproducible MIC values under standardized conditions. Additionally, Menon^[^ ^11^ ^]^ reported inhibitory activity of lupeol against MRSA at 50 μg.mL^−1^, with subinhibitory effects on mecA gene expression observed at 20 μg.mL^−1^, suggesting that lupeol's antimicrobial activity may vary based on the bacterial strain and the specific assay employed.

**Figure 1:**
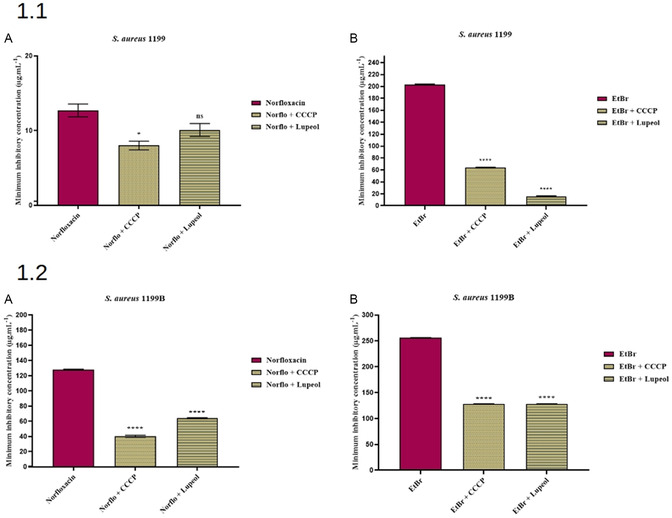
1.1. Evaluation of *NorA* efflux pump inhibition by lupeol in *S. aureus* *strains A) 1199 and B) 1199B 1.2. Lupeol was tested in combination with norfloxacin (A) and ethidium bromide (EtBr) (B). Data were analyzed using one‐way ANOVA followed by Bonferroni's post hoc test. ***p < 0.0001 versus *control (in MIC reduction); ns = not significant* versus *control.*

In **Figure** **1** 1.1A, it can be observed that the combination of lupeol with norfloxacin against the SA1199 strain resulted in a reduction of the antibiotic's MIC to 10.2 μg mL^−1^, compared to 12.7 μg mL^−1^ when norfloxacin was used alone. Although this reduction indicates an enhancement of norfloxacin activity by lupeol, the combination of norfloxacin with the standard efflux pump inhibitor CCCP showed even greater efficacy, reducing the MIC to 8 μg mL^−1^.

In Figure 1 1.1B, the MIC of ethidium bromide (EtBr) decreased from 203.2 μg mL^−1^ (control) to 16 μg mL^−1^ when combined with lupeol. This corresponds to an ≈12.7‐fold reduction, suggesting a potential synergistic interaction and likely inhibition of efflux activity. For comparison, the MIC of EtBr in combination with the standard efflux pump inhibitor CCCP was 64 μg mL^−1^, indicating that lupeol exhibited a stronger potentiating effect under the tested conditions.

However, in the SA1199 strain, the combination of lupeol with norfloxacin did not result in a significant MIC reduction compared to norfloxacin alone. This absence of apparent synergy may be due to a chemical interaction between lupeol and norfloxacin, possibly involving chelation, which could impair the antimicrobial activity of the antibiotic.

Figure 1 1.2. presents the MIC data for the SA1199B strain, which is known to overexpress the NorA efflux pump. In Figure 1 1.2A, the MIC of norfloxacin decreased from 256 μg mL^−1^ (control) to 64 μg mL^−1^ when coadministered with lupeol, representing a fourfold reduction. For comparison, the MIC in the presence of CCCP was 40.3 μg mL^−1^, indicating that although lupeol was less effective than CCCP, it still contributed to significant efflux inhibition.

In Figure 1 1.2B, the combination of lupeol with EtBr resulted in an MIC of 128 μg mL^−1^, which was equivalent to that observed with CCCP and half of the value obtained in the untreated control (256 μg mL^−1^). These findings support the hypothesis that lupeol interferes with the NorA efflux mechanism.

Overall, the MIC reductions observed for both norfloxacin and ethidium bromide in the SA1199B strain—especially when resembling the patterns obtained with CCCP—suggest that lupeol may function as an inhibitor of the NorA efflux pump. The consistency of MIC reductions across replicates and with different substrates (EtBr and norfloxacin) indicates good experimental reproducibility.

### 2.2 Membrane Permeability Assessment

**Figure 2:**
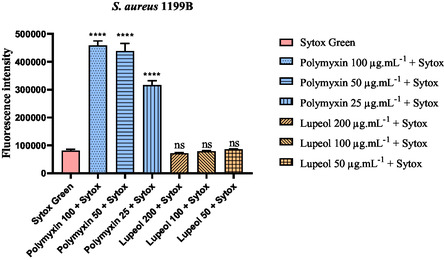
Evaluation of lupeol's effect on the membrane permeability of *S. aureus* *1199B. Results are expressed as mean fluorescence intensity. Statistical analysis was performed using one‐way ANOVA followed by Tukey's post hoc test. ***p < 0.0001 versus *Sytox Green; ns = not significant* versus *Sytox Green.*

When assessing the ability of lupeol to induce permeabilization of the cytoplasmic membrane in the SA1199B strain (**Figure** **2**), no significant increase in fluorescence intensity was detected compared to the negative control (untreated cells). This result indicates that lupeol does not compromise the membrane integrity of *Staphylococcus aureus* under the tested conditions.

In contrast, the positive control treated with polymyxin B exhibited a marked increase in fluorescence intensity, confirming membrane disruption and validating the experimental setup. The clear distinction between the negative and positive controls further supports the reliability and reproducibility of the assay.

### 2.3 Fluorescence Evaluation of Ethidium Bromide

**Figure 3:**
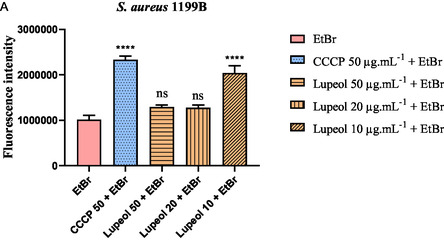
Evaluation of *NorA* efflux pump inhibition in *S. aureus* *1199B based on fluorescence emission. Bacterial cells were treated with lupeol at concentrations of 50, 20, and 10 μg mL^−1^. EtBr = ethidium bromide. Statistical analysis: ****p < 0.0001 versus EtBr; *p < 0.01 versus *EtBr; ns = not significant* versus *EtBr.*

In the ethidium bromide accumulation assay using the *Staphylococcus aureus* SA1199B strain (**Figure** **3**), lupeol at a concentration of 10 μg.mL^−1^ promoted a significant increase of 100% in fluorescence emission compared to the negative control (EtBr alone), indicating enhanced intracellular accumulation of the dye. This suggests inhibition of efflux pump activity. Lupeol at concentrations of 20 μg.mL^−1^ and 50 μg.mL^−1^ also led to increases in fluorescence, though to a lesser extent—27% and 29%, respectively.

The positive control, CCCP (carbonyl cyanide m‐chlorophenyl hydrazone), similarly induced a 100% increase in fluorescence, confirming the expected inhibition profile of the NorA efflux pump and validating the assay conditions. These results support the hypothesis that lupeol interferes with NorA‐mediated efflux, particularly at lower concentrations, and may act as an efflux pump inhibitor in *S. aureus*.

### 2.4 Molecular Docking

**Figure 4:**
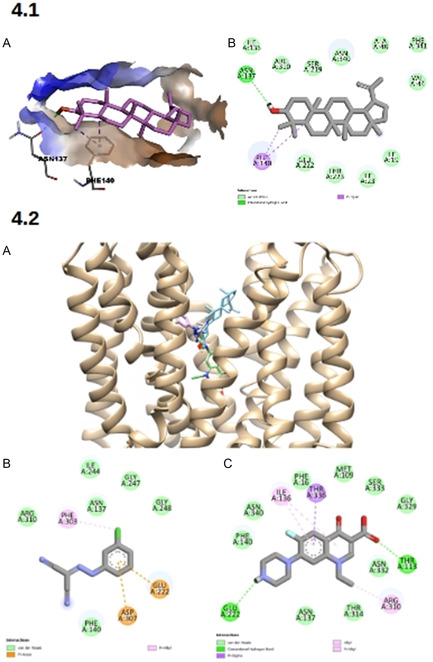
[4.1]. Lupeol conformation within the A) NorA binding site and B) molecular interactions established with *NorA*. [4.2]. *Molecular docking results for NorA*: (A) binding site location of ligands (lupeol in blue, CCCP in pink, and norfloxacin in green); (B) interactions established by CCCP; and C) interactions established by norfloxacin.

The binding energy of lupeol with the NorA efflux pump model was −9.540 kcal mol^−1^, showing higher affinity than CCCP, which presented an energy of −7.112 kcal mol^−1^. In addition to van der Waals interactions with residues Ala48, Arg310, Asn340, Glu222, Ile19, Ile23, Ile136, Phe341, Ser219, Thr223, and Val44, lupeol also interacted with two additional residues: Asn137, with which it formed a hydrogen bond, and Phe140, which participated in two π‐sigma interactions (**Figure** **4**).

CCCP, in turn, also performed van der Waals interactions (with Arg310, Asn137, Gly247, Gly248, Ile244, and Phe140) and interacted with NorA through π‐anion (Asp307 and Glu222) and *π*‐alkyl (Phe303) interactions (Figure 4). Although the binding energy of CCCP is less negative than that of lupeol, this inhibitor showed better results in the association with norfloxacin in vitro.

Molecular docking with the fluoroquinolone norfloxacin (binding energy = 8.815 kcal mol^−1^) showed that, although the best conformations of the ligands occur in nearby regions, only CCCP interacts directly with a residue shared with the antibiotic, the Glu222 residue (Figure 4). This is the main factor that justifies the greater efficacy of CCCP in potentiating norfloxacin.

The findings of the present study indicate that lupeol exhibits intrinsically limited antibacterial activity, with no relevant clinical significance. This result is in line with the data reported by Rosandya and collaborators,^[^ ^12^ ^]^ who observed low efficacy of the compound against different bacterial strains. Similarly, Puisais and Cock^[^ ^13^ ^]^ highlighted the variability of the results available in the literature regarding the antimicrobial activity of lupeol, suggesting that such divergences reflect methodological gaps and the need for more systematic and robust investigations.

Although lupeol exhibited a high MIC (≥1024 μg.mL^−1^), which suggests low direct antibacterial activity, the results demonstrate that it plays a role in modulating resistance, acting as a functional inhibitor of the NorA efflux pump. This finding is particularly relevant, since active efflux mechanisms such as that mediated by NorA represent one of the main determinants of phenotypic resistance in *Staphylococcus aureus*, reducing the intracellular concentration of antibiotics and compromising their therapeutic efficacy. The observed effect is consistent with the study by Ramalhete et al.,^[^ ^14^ ^]^ who demonstrated that triterpenes isolated from *Momordica balsamina* are capable of inhibiting bacterial efflux pumps, an effect attributed to the structural conformation of these compounds and their affinity for allosteric domains of the transport protein.

The molecular docking analysis performed in this work reinforces the plausibility of the proposed mechanism of action. Lupeol showed stable interaction with functional residues previously associated with *NorA* pump activity, notably Asn137 and Phe140. These interactions, of the hydrophobic and hydrogen bonding type, were described by Kumar and Tudu^[^ ^15^ ^]^ as recurrent in natural compounds with an inhibitory profile against *NorA*, which confers predictive validity to the molecular model used and supports the modulating potential of Lupeol.

Additionally, the docking data corroborate the observations of Işik and Serçinoğlu,^[^ ^16^ ^]^ who described the functional importance of residues such as Asn137, Phe140, and Glu222 in the conformation and performance of NorA. The latter, in particular, was associated with the activity of classical inhibitors such as CCCP, whose high affinity for this site may justify its superiority in the potentiation of norfloxacin. The comparison between the interaction profile of lupeol and CCCP reinforces the hypothesis that lupeol, although less potent, shares critical interaction sites with established inhibitors, which is indicative of a convergent mechanism of action.

To evaluate the stability of the lupeol–NorA complex over time, molecular dynamics simulations were conducted, and the root‐mean‐square deviation (RMSD) values of the ligand within the binding pocket were monitored. The RMSD analysis showed a stable trajectory with fluctuations remaining below 2.5 Å throughout the simulation time, suggesting a consistent and stable binding conformation.^[^ ^16^, ^17^ ^]^

This finding supports the hypothesis that the interactions observed in docking are not merely artifacts of static modeling, but are maintained in a dynamic environment, indicating a reliable and durable interaction.^[^ ^17^, ^18^ ^]^

In contrast, the CCCP–NorA complex, although showing a less negative docking energy, also demonstrated stability in molecular dynamics, with RMSD values fluctuating around 2.0 Å. Interestingly, CCCP interacts with Glu222—an essential residue also involved in the binding of norfloxacin—reinforcing its role in modulating antibiotic potency. The absence of direct interaction between lupeol and Glu222 may partially explain its lower efficacy as a potentiator, despite its higher theoretical binding affinity.^[^ ^15^ ^]^

Together, these findings highlight the importance of evaluating both binding energy and conformational stability when predicting functional inhibition of efflux pumps. While lupeol displays promising interactions with NorA, its inability to stabilize interactions with key residues involved in norfloxacin recognition may limit its synergy with antibiotics.^[^ ^17^, ^18^ ^]^

Therefore, although lupeol does not exhibit relevant antibacterial activity in isolation, its ability to modulate the activity of efflux pumps gives it potential pharmacological value as a therapeutic adjuvant. Its triterpene structure, combined with its affinity for *NorA*‐determining residues, suggests that this natural compound may contribute to the phenotypic reversal of antimicrobial resistance, configuring it as a promising tool in the development of therapeutic adjuvants aimed at combating infections caused by multidrug‐resistant strains of *S. aureus*.

## 3 Conclusions

Bacterial resistance to conventional antibiotics is one of the main therapeutic obstacles today, requiring the identification of new strategies capable of mitigating adaptive mechanisms, such as the action of efflux pumps. In this context, natural compounds have emerged as promising alternatives, especially due to their structural diversity and ability to modulate diverse molecular targets.

The present study demonstrated that the triterpene lupeol, although not presenting direct antibacterial activity at clinically relevant concentrations, exhibits significant inhibitory potential on the *NorA* efflux pump in *Staphylococcus aureus*. The in vitro functional analysis was corroborated by molecular modeling, which revealed high affinity of lupeol for critical residues of the protein, surpassing the positive control CCCP in terms of binding energy. These findings support the hypothesis of functional modulation of the pump, with potential impact on the antimicrobial efficacy of *NorA* substrate agents.

Thus, lupeol appears as a relevant candidate for the design of therapeutic adjuvants aimed at reversing resistance in multidrug‐resistant strains. Future studies should deepen the elucidation of the molecular mechanisms involved, as well as validate its performance in clinical models, consolidating its applicability in tackling antimicrobial resistance, one of the most urgent challenges in contemporary medicine.

## 4 Experimental Section

4.1

4.1.1

##### Obtaining Lupeol

Lupeol (C_30_H_50_) used in the research was purchased from Sigma–Aldrich by Prof. Dr. José Maria Barbosa‐Filho and kindly provided to our research group.

##### Bacterial Strains

*Staphylococcus aureus* SA1199 and SA1199B strains, both donated by Prof. Dr. Glenn Kaatz (Wayne State University School of Medicine) and Prof. Dr. Simon Gibbons (University College London), were used for the analyzes. The strains were initially reconstituted from their lyophilized state in sterile brain heart infusion (BHI) broth, incubated at 37 °C for 24 h and subsequently plated on Mueller‐Hinton agar for activation and obtaining viable colonies. Strain SA1199 was known to overexpress the *NorA* efflux pump and was widely used in screening assays for efflux inhibitors. Strain SA1199B represents a hypersensitive variant with basal levels of *NorA* expression, serving as a comparative control to evaluate the activity of efflux modulators.

##### Determination of Minimum Inhibitory Concentration

The MIC of lupeol was determined according to CLSI guidelines,^[^ ^19^ ^]^ using the broth microdilution method in sterile 96‐well microplates. Bacterial strains were grown on agar plates for 24 h at 37 °C. Inocula were prepared in sterile saline from isolated colonies and adjusted to a 0.5 McFarland standard. Lupeol was previously dissolved in dimethyl sulfoxide (DMSO) at a concentration of 1024 μg.mL^−1^. Each well received 100 μL of the lupeol solution and 100 μL of the bacterial suspension, for a final volume of 200 μL. The remaining volume was filled with BHI broth as the culture medium. Growth control wells containing only inoculated medium, and sterility control wells containing only BHI broth were included. Plates were incubated at 37 °C for 24 h. After incubation, 20 μL of resazurin solution was added to each well to assess cell viability.

##### Efflux Pump Inhibition Assessment

The efflux pump inhibitory activity was evaluated using lupeol at a subinhibitory concentration (MIC/8), as well as standard efflux pump inhibitors, following protocols adapted from CLSI guidelines^[^ ^19^ ^]^ and the method described by Tintino et al.^[^ ^20^ ^]^ Test solutions were prepared with 10% of a standardized bacterial inoculum (corresponding to 0.5 on the McFarland scale), diluted in BHI broth and supplemented with lupeol or efflux modulators. This mixture was distributed into sterile 96‐well microdilution plates, each well containing 100 μL of serially diluted antibiotic or ethidium bromide (EtBr) at previously determined concentrations. To ensure experimental reliability, the following controls were included: a positive growth control, consisting of bacterial inoculum and BHI broth only, to confirm cell viability; a sterility (negative) control, containing only BHI broth, to check for contamination; a control with antibiotic or EtBr alone, to determine their baseline activity; and a control with the modulator alone (lupeol or standard inhibitor) at a subinhibitory concentration, to assess any direct effect on bacterial growth. Additionally, combinations of antibiotic or EtBr with either lupeol or the standard efflux inhibitor were tested to investigate potential synergistic interactions or efflux pump inhibition. The plates were incubated at 37 °C for 24 h. After incubation, resazurin was added to each well to assess cell viability. Efflux pump inhibition was inferred based on two criteria: 1) reduction in the MIC of the antibiotic, indicating enhanced antimicrobial efficacy in the presence of the modulator and 2) increased EtBr fluorescence, suggesting intracellular accumulation of the compound due to efflux pump inhibition.

##### Membrane Permeability Assessment

The SYTOX Green dye was used. The *S. aureus* 1199B inocula were prepared according to the McFarland scale standard 0.5 and distributed in the 96‐well black plate. Lupeol was added until final concentrations of 200 μg mL^−1^, 100 μg mL^−1^, and 50 μg mL^−1^ were obtained. Polymyxin B was used as a positive control at final concentrations of 100 μg mL^−1^, 50 μg mL^−1^, and 25 μg mL^−1^, and in the negative control only phosphate‐bufferid saline (PBS) was added to the inoculum. The plates were incubated for 1 h. 100 μL of SYTOX Green was added to the wells until the final concentration of 1 μM was obtained, then the plates were incubated for 30 min. The reading was performed on the Cytation 1 fluorescence reader, BioTek (Winooski, VT, USA), and Gen5 3.11 software. A 485 nm excitation and 528 nm emission filter were used. The tests were performed in triplicate^[^ ^21^ ^]^

##### Fluorescence Evaluation of Ethidium Bromide

The *S. aureus* 1199B strains were preseeded and then incubated in a bacteriological incubator at 37 °C. The bacterial inoculum was prepared in PBS buffer according to the 0.5 index of the McFarland scale. Solutions containing bacterial inoculum and lupeol at concentrations of 10, 20 or 50 μg.mL^−1^ were prepared. CCCP 50 μg.mL^−1^ was used as a positive control and the negative control consisted only of PBS and bacterial inoculum. These solutions were incubated for 1h30min. Subsequently, EtBr 100 μg.mL^−1^ was added to all solutions and kept again in a bacteriological incubator. Then, the solutions were centrifuged at 10 000 rpm for 2 min and washed with PBS, discarding the supernatant until the EtBr and remaining substances were removed. The pellet was dissolved in PBS and distributed in black microplates. The reading was performed with a fluorescence microplate reader (Cytation 1, BioTek, Winooski, VT, USA), and Gen5 3.22 software, using excitation at 530 nm and emission wavelength at 590 nm. The assay was performed in triplicate.^[^ ^22^ ^]^

##### Molecular Docking

The models of the *NorA S. aureus* efflux pumps were constructed by homology, from the sequences available in the UniProt database (codes P0A0J7 and Q2G140, respectively), with the aid of the SWISS‐MODEL server.^[^ ^23^, ^24^ ^]^ The elaboration of the ligand structures was carried out in the MarvinSketch software (Chemaxon, version 23.14), with energy minimization through the use of the MMFF94 force field (from the English Merck Molecular Force Field), included in the Open Babel software, version 2.4.1.^[^ ^25^ ^]^

The preparation of receptors and ligands took place in AutoDockTools 1.5.7,^[^ ^26^ ^]^ with the addition of hydrogen atoms (mixing the nonpolar ones), partial Gasteiger charges and flexibility. For the receptors, important residues for their functioning were kept flexible, namely residues 16, 20, 23, 137, 140, 222, 255, 303, 306, 307, and 336, for NorA. We performed the molecular docking analyzes using AutoDock Vina 1.2.5,^[^ ^27^ ^]^ centering the grid box in the respective central cavities and using dimensions (*x*, *y*, *z*) of 30 Å × 30 Å × 30 Å (NorA), which consider the arrangement of the aforementioned residues in the respective targets. The selection of the best results (conformations) was based on the lowest binding energies, calculated in Kcal/mol. UCSF Chimera and BIOVIA Discovery Studio^[^ ^28^ ^]^ were used to visualize the results.

The docking experiments were validated following the study by Martin et al.,^[^ ^12^ ^]^ which performed double‐checking and considered the limit of 2.0 Å for RMSD.

##### Statistical Analysis

For statistical analysis, Graphpad Prism software, version 8.01, was used, applying two‐way ANOVA followed by the Bonferroni posttests, considering statistical significance *p* < 0.01.

## Conflict of Interest

The authors declare no conflict of interest.

## Data Availability

The data that support the findings of this study are available from the corresponding author upon reasonable request.
